# Association of vWA and TPOX Polymorphisms with Venous Thrombosis in Mexican Mestizos

**DOI:** 10.1155/2014/697689

**Published:** 2014-08-31

**Authors:** Marco Antonio Meraz-Ríos, Abraham Majluf-Cruz, Carla Santana, Gino Noris, Rafael Camacho-Mejorado, Leonor C. Acosta-Saavedra, Emma S. Calderón-Aranda, Jesús Hernández-Juárez, Jonathan J. Magaña, Rocío Gómez

**Affiliations:** ^1^Departamento de Biomedicina Molecular, Centro de Investigación y de Estudios Avanzados del Instituto Politécnico Nacional (Cinvestav-IPN), Avenida Instituto Politécnico Nacional 2508, Colonia San Pedro Zacatenco, 07360 México, DF, Mexico; ^2^Unidad de Investigación Médica en Trombosis, Hemostasia y Aterogénesis, Instituto Mexicano del Seguro Social, México, DF, Mexico; ^3^Laboratorio Biología Molecular Diagnóstica (BIMODI), Querétaro, QRO, Mexico; ^4^Departamento de Toxicología, Centro de Investigación y de Estudios Avanzados del Instituto Politécnico Nacional (Cinvestav-IPN), Avenida Instituto Politécnico Nacional 2508, Colonia San Pedro Zacatenco, 07360 México, DF, Mexico; ^5^Laboratorio de Medicina Genómica, Departamento de Genética, Instituto Nacional de Rehabilitación, Secretaría de Salud, México, DF, Mexico

## Abstract

*Objective*. Venous thromboembolism (VTE) is a multifactorial disorder and, worldwide, the most important cause of morbidity and mortality. Genetic factors play a critical role in its aetiology. Microsatellites are the most important source of human genetic variation having more phenotypic effect than many single nucleotide polymorphisms. Hence, we evaluate a possible relationship between VTE and the genetic variants in von Willebrand factor, human alpha fibrinogen, and human thyroid peroxidase microsatellites to identify possible diagnostic markers. *Methods.* Genotypes were obtained from 177 patients with VTE and 531 nonrelated individuals using validated genotyping methods. The allelic frequencies were compared; Bayesian methods were used to correct population stratification to avoid spurious associations. *Results.* The vWA-18, TPOX-9, and TPOX-12 alleles were significantly associated with VTE. Moreover, subjects bearing the combination vWA-18/TPOX-12 loci exhibited doubled risk for VTE (95% CI = 1.02–3.64), whereas the combination vWA-18/TPOX-9 showed an OR = 10 (95% CI = 4.93–21.49). *Conclusions.* The vWA and TPOX microsatellites are good candidate biomarkers in venous thromboembolism diseases and could help to elucidate their origins. Additionally, these polymorphisms could become useful markers for genetic studies of VTE in the Mexican population; however, further studies should be done owing that this data only show preliminary evidence.

## 1. Introduction

Venous thromboembolism (VTE) is a multifaceted disorder with high levels of morbidity, mortality, and recurrence worldwide [1]. Clinically, it is defined by deep vein thrombosis, pulmonary embolism, or both [2]. VTE exhibits a multifactorial aetiology and several risk factors such as age, trauma, hormonal misbalance, immobility, hypercoagulable state, thrombophilic defects, and hypertension are involved in its development [3]. Additionally, genetic factors play a critical role in the aetiology, and, consequently, the identification of predictive biomarkers is an active area, which may contribute to its diagnosis [4]. Microsatellites, also named short tandem repeats (STRs), are the most important source of human genetic variation [5]. STRs are located in 17% of the human coding genes, exhibiting a wide distribution in regulatory regions affecting the transcription and gene expression [6]. Hence, STRs have been linked to disease phenotypes, principally, in neuromuscular and neurodegenerative disorders [7]. Currently, STRs have been also related to complex diseases such as cancer, diabetes, and cardiovascular diseases (CVD), suggesting that STRs have more phenotypic effect than many single nucleotide polymorphisms (SNPs) [5, 6, 8].

Within the combined DNA index system (CODIS), five STRs are located within genes: TH01 (human tyrosine hydroxylase), TPOX (human thyroid peroxidase), vWA (von Willebrand factor), CSF1PO (c-fms protooncogene for CSF-1 receptor gene), and FGA (human alpha fibrinogen). Some of these, such as vWA, FGA, and TPOX, have been associated with thrombotic events. Von Willebrand factor (vWF) is a glycoprotein produced by vascular endothelium and platelet [9]. As well, vWF is essential for haemostasis and promotes thrombosis by platelet adhesion and aggregation and clinically acts as a biomarker of CVD [10]. Synthesis and secretion of vWA are regulated genetically and, as of now, more than 19 SNPs in introns regions have been associated with vWF antigen levels [11]. In addition, a complex STR marker located in intron 40 was associated with diseases related to coagulation [12]. With respect to fibrinogen, this is considered the risk factor for CVD, and genetic variation has been associated with fibrinogen levels as well as fibrin network structure [13, 14]. Therefore, polymorphisms in FGA have been associated with a stroke risk as well as an increased risk of VTE [10, 15, 16]. Ultimately, several reports suggest that coagulation abnormalities and vascular endothelial dysfunctions are correlated with thyroid peroxidase (TPOX) activity [17]. As well, thyroid levels have been associated with a thromboembolic potential suggesting that TPOX could contribute to VTE [18]. The search of biomarkers associated with thrombosis risk aims to contribute to and identify molecular bases of this complex disease using polymorphic markers [4]. In addition, the implementation of genetic predictive diagnostic tests could be used for the instauration of preventive treatments to avoid the onset of illness. However, genotype-phenotype association studies have been commonly focused on single nucleotide polymorphisms (SNPs) underestimating markers of hypervariable polymorphisms [19]. Thus, the major purpose of the present paper is to seek a possible relationship between VTE and the combined effect of more than one genetic variant in vWA, FGA, and TPOX microsatellites in order to identify possible susceptibility markers. We employed a case-control study using three controls for each case. Our findings suggest that allele 18-vWA, as well as 9-TPOX and 12-TPOX, could be related to thrombosis risk in our population. This association is not an artificial effect, since it was maintained even after population stratification correction. Our data corroborates that STR analysis is a good strategy for developing markers to elucidate the origins of many human genetic diseases.

## 2. Materials and Methods

### 2.1. Subject Selection

We analysed the genetic data of 708 (1416 chromosomes) unrelated individuals born in Mexico who had at least three generations of ancestors born in this country. The studied population was subdivided in two groups: the first group (cases) consisted of 177 individuals (98 women and 79 men), with unprovoked venous thrombosis disease recruited at the Thrombosis, Haemostasis and Atherogenesis Medical Research Unit (Unidad de Investigación Médica en Trombosis, Hemostasia y Aterogénesis (UIMTHA)) of the Mexican Institute of Social Security (IMSS, initials in Spanish) in Mexico City. Unprovoked VTE was defined as VTE that occurred in the absence of trauma, surgery, immobilisation during three or more days, and no evidence of malignant disease for the past five years. Clinical suspicion of unprovoked venous thrombosis was confirmed in all patients by a combination of at least two of the following tests: a positive D-dimer, contrast venography, compression ultrasonography, ventilation/perfusion scanning, or chest computed tomography scan. All patients had one (39%) or more (61%) unprovoked episodes of venous thrombosis. For the whole group, a family history of venous thrombosis was found in 32% of the patients while 15% of them had a family history of arterial thrombosis at young age (<45 years old). In 29% of the patients, there was a history of death before 45 years old in at least one direct relative due to an arterial or venous thrombotic event. The second group (controls), composed of 531 (three controls by each case) nonrelated healthy individuals (239 women and 292 men), was recruited by BIMODI laboratory. This population is a representative group of the Mexican mestizos as has been described previously by our research group [20]. Each individual signed an informed consent validated by the Ethics Committee of the BIMODI Research Unit and the UIMTHA. Genealogical data were also obtained from each person to ensure that the individuals were unrelated through at least three generations. Trained personnel interviewed all individuals in order to obtain the baseline information (Table 1).

### 2.2. Polymorphisms Analysis

Genomic DNA was extracted from peripheral blood leukocytes using QIAamp DNA mini kit (Qiagen, Düsseldorf, Germany). Polymerase chain reaction (PCR) was performed with oligonucleotide primers previously reported by Kimpton et al. [21], Anker et al. [22], and Promega Corporation [23]. Approximately 10 ng target DNA was amplified using a multiplex reaction standardized in our laboratory. The reaction was standardized at 6 *μ*L total volume, containing 0.015 *μ*M primers, 1X reaction buffer with NH_4_SO_2_, 2.5 mM MgCl_2_, 200 *μ*M of each nucleotide (Thermo Fisher Scientific, Suwanee, GA, USA), 1 M betaine (Sigma-Aldrich, St. Louis, MO, USA), and 1 U Taq DNA polymerase (Thermo Fisher Scientific, Suwanee, GA, USA). The thermocycling procedure consisted of 35 cycles of denaturation at 94°C for 30 sec, annealing at 55°C for 1 min, and extension at 72°C for 1 min, followed by a final extension step of ten minutes at 72° C. The resulting amplicons were analysed by capillary electrophoresis on the ABI Prism 3130XL Genetic Analyser using the GeneMapper ID v. 3.2. software (Applied Biosystems, Carlsbad, CA, USA). We also used the control DNA 007 (Applied Biosystems, Carlsbad, CA, USA) as a validated internal control.

### 2.3. Statistical Analysis

Allele and genotype frequencies were performed using Arlequin v.* 3.1* software [24]. Hardy-Weinberg expectation (HWE) was calculated by applying Weir and Cockerham *F* Statistic (*F*_IS  w&C_), using Genètix v.* 4.05.2* program [25]. The levels of significance were determined empirically with 10,000 permutations. To examine allelic and genotype frequencies, differentiation between pair of groups, defined by disease status, was computed by the Chi-squared test (*χ* ^2^) using the Epi Info v.* 7* software [26]. In order to assess the degree of dependence of disease on genotypes, we calculated odds ratio (OR) using Epi Info v.* 7* software. The associations between alleles of STR's polymorphisms and the risk to develop VTE were estimated by OR through simple and multiple logistic regression models. Multivariate model was adjusted using confounders such as gender, age, smoking status, and family history, with the STATA 10.0 software package (Stat Corporation, College Station, TX, USA). Ninety-five percent confidence intervals (95% CI) and *P* values are reported for the OR.

### 2.4. Genetic Structure Analysis

Population genetic structure was inferred previously applying a model based on Bayesian statistics, using the Structure v.* 2.3.3* software [27], which uses genotypic correlations among unlinked markers to learn about the structure of the population under study and the genetic background of the individuals. The program uses the Markov chain Monte Carlo (MCMC) method to estimate the number of subpopulations, allele frequencies in each subpopulation, and the *q* value for each sampled individual. All runs were performed using the conditions previously reported by Gómez et al. [28].

### 2.5. Correction of Admixture

STRAT v.* 1.1* software (University of Oxford, Oxford, UK) was used to correct the impact of admixture over results avoiding spurious association [29]. This software is a companion program to Structure v.* 2.3.3* software [27]. Previous results obtained by our research group demostrated that Mexican mestizo populations shows trihybrid ancestry as a consequence correction for three subpopulations (*k* = 3) was used [20].

## 3. Results 

### 3.1. Descriptive Statistics

The distribution of allele frequencies of three loci and descriptive statistics for both studied populations are shown in Table 2. Results demonstrated that some allele frequencies in vWA, TPOX, and FGA presented differences when comparing case and control populations. In regard to genotypic frequencies, the genotypes, TPOX-9,9 (0.175), vWA-16,17 (0.153), TPOX-9,12 (0.147), vWA-16,18 (0.113), vWA-23,25 (0.051), FGA-20,25 (0.045), and FGA-21,25 (0.045), were the most frequent in cases group. Insofar as control group, the most frequent genotypes were TPOX-8,8 (0.275), TPOX-8,11 (0.224), vWA-16,17 (0.194), vWA-16,18 (0.105), vWA-16,16 (0.087), FGA-22,24 (0.060), FGA-24,25 (0.051), and FGA-21,24 (0.045) (see Supplemental Material available online at http://dx.doi.org/10.1155/2014/697689).

On the other hand, the descriptive statistical analysis showed a Hardy-Weinberg departure (HWD) in vWA and TPOX in the thrombotic population. Interestingly, this HWD was related to an excess of homozygotes (*F*_IS_ > 0) and is shown only in cases group: vWA (*F*_IS_ = 0.122, *P* = 0.0005) and TPOX (*F*_IS_ = 0.242, *P* = 0.0001) (Table 2). On the other hand, the control population was in agreement with HWE even after Bonferroni correction (*P* ≤ 0.016). All locus combinations showed linkage equilibrium adjusting *P* value for 5% of nominal level (*P* = 0.0000480).

### 3.2. Frequency Differences between Cases-Controls

In order to detect whether these frequency differences could be related to a possible association with venous thrombosis, we determined the statistical significance. All data was adjusted using Bonferroni correction (*P* ≤ 0.016). The possible risk alleles exhibiting significant differences are shown in Table 3. Even though all seven alleles in Table 3 exhibited significant statistical differences (*P* ≤ 0.001), only vWA-18, TPOX-9, TPOX-12, FGA-26, and FGA-27 presented a possible association with thrombosis due to OR as well as confidence intervals. Consequently, only these loci were considered possible candidates in relation to the venous thrombosis phenotype. With respect to genotype frequencies, significant differences were found in the following genotypes: vWA-14,16 (*P* = 0.004), vWA-18,18 (*P* ≤ 0.0001), TPOX-9,9 (*P* ≤ 0.0001), TPOX-9,12 (*P* ≤ 0.000), TPOX-12,12 (*P* ≤ 0.0001), FGA-21,27 (*P* = 0.048), FGA-22,26 (*P* = 0.01), FGA-22,27 (*P* ≤ 0.001), FGA-23,27 (*P* ≤ 0.01), FGA-25,27 (*P* = 0.002), FGA-26,26 (*P* = 0.02), and FGA-26,28 (*P* = 0.02) (Supplemental Material).

### 3.3. Admixture Correction

Focused on identifying truly genetic associations, we corrected the stratification in the population; this parameter is the principal confusing factor that could lead to spurious association [30, 31]. To avoid it, we validated our case-control study in the presence of a population structure using the trihybrid model (*k* = 3) previously reported by our research group in the Mexican mestizo population [20]. After the admixture correction, vWA and TPOX loci maintained the statistical differences with *P* = 0.043 and *P* ≤ 0.0001, respectively (Table 3). This evidence suggests that the proportion of individuals carrying a risk allele, either in heterozygote genotype or as homozygote, can be different among case and control populations. Consequently, we did an analysis comparing these frequencies in both populations (Table 4). Our results showed that the genetic dose of the venous thrombosis associated allele vWA-18 was 22% in cases and 14% in controls (OR = 1.51, 95% CI = 1.04–2.20, *P* = 0.0232), whereas the genetic doses of TPOX-9 and TPOX-12 thrombosis associated alleles were ≈43% in cases and ≈6% in controls (OR = 17.06, 95% CI = 11.06–26.36, *P* ≤ 0.0001), and ≈31% in cases and 12% in controls (OR = 2.3, 95% CI = 1.6–3.3, *P* ≤ 0.0001), respectively.

### 3.4. Association of Alleles and Genotypes with VTE by Adjusted Model

In agreement with Table 4, we analysed the presence of alleles and genotypes of each of the candidate polymorphisms.  Table 5 depicts the determination of risk to present VTE in the Mexican population taking into account some confounders, such as age, gender, smoking status, and family history. Under this analysis, we found that individuals carrying the vWA-18 allele displayed a high risk to present VTE (OR = 2.32). Consequently, the homozygote genotype (18, 18) has a higher susceptibility for VTE in comparison with the heterozygote genotype. Likewise, subjects carrying the TPOX-9 allele have higher risk to present VTE than those bearing the other allele (OR = 10.66). In concordance, subjects with the homozygote genotype to 9 allele (9,9), have higher association than those bearing the 9 heterozygous genotypes. Interestingly, the presence of homozygote states for susceptibility alleles in vWA (8%) and TPOX (17.5%) was more frequent in patients with VTE in comparison with the controls (vWA, 0.75%; TPOX, 0.37%). These results showed the important contribution of these alleles to the development of VTE. Finally, the allele 12 of TPOX marker did not show an important implication with VTE, unless it is present in its homozygous form. These results suggest that vWA-18 allele, TPOX-9 allele, and TPOX-12,12 genotype act as risk factors for VTE (Table 5).

### 3.5. Multiloci Genotype Analysis

Finally, we combined the risk alleles in both loci (multiloci genotypes) to find a possible risk combination (Table 6). Our results indicated that the combination of allele 18 (vWA) with allele 9 (TPOX) as well as 18 (vWA) with 12 (TPOX) yielded a significant difference (OR = 10.21, 95% CI = 4.93–21.49, *P* ≤ 0.0001; OR = 1.94, 95% CI = 1.02–3.64, *P* < 0.05), suggesting that this combination could be associated with an increase in the venous thrombosis risk.

## 4. Discussion

Thrombosis is a complex disease associated with genetic and environmental factors. As some studies have previously reported, different genes participate in procoagulant and anticoagulant pathways and have been studied in different populations. These studies showed that some SNP polymorphism markers in* PAI-1*,* Lp(a)*,* JAK2*,* PON-1*,* MTHFR*,* ABO*, and* vWF* loci, among others [23–26], could be associated with thrombotic disease [32–35]. Additionally, several studies related to clinical aspects support some of these hypotheses [36].

Using population genetics, we have shown evidence with a strong association between vWA (allele 18) and venous thrombosis (OR = 1.77, 95% CI = 1.29–2.43, *P* = 0.0001) even after admixture correction and adjusted confounders analysis. These findings are supported by previous studies which exhibited a strong relationship between serum levels of von Willebrand factor and vWA polymorphic variants (SNP and (GT)n in promoter region), where both contributed to the risk of CVD [37, 38]. Therefore, vWA polymorphism may be involved in thrombosis disease owing to this gene that has a central role in blood coagulation systems and it is also the major mediator of platelet adhesion as well as the carrier for the coagulation factor VIII [12]. Interestingly, we also found that the TPOX gene, which presents a STR [AATG]n within intron 10, could be associated with venous thrombosis. This STR has not been used as a genetic marker of CVD; however, clinical reports relate high TPOX levels with hyperthyroidism, supporting a possible association with thrombosis, since hyperthyroidism is related to hypercoagulable states [18, 39]. Our results showed that alleles (AATG) ≥9 tandem repeats present strong association (OR = 7.22, 95% CI = 5.12–10.18, *P* ≤ 0.0001; OR = 1.93, 95% CI = 1.39–2.68, *P* ≤ 0.0001, resp.) with venous thrombosis. Mainly, the presence of the allele 9 and both genotypes in homozygous and heterozygous form could be important markers for the risk for developing VTE. Similarly, the allele 12 in its homozygous form showed an increased risk for VTE.

Both STR markers maintained a significant association even after population stratification adjustment suggesting that demographic events in the Mexican mestizo population such as intercontinental migrations, bottlenecks, high demographic expansions, and the youth of the population (only 10–15 generations) are not confounder factors. In addition, the HWD found in cases and not in the controls could support the possibility of risk alleles [40, 41]. These findings are principally shown in case-control studies where the excess of homozygous breaks the random mating. As a consequence, this HWD could be attributed to the disease association [42, 43]. Consequently, the strength of the association is enhanced. As well, it is worthwhile to mention that the homozygote state of the risk alleles (vWA-18 and TPOX-9) seems evident, significantly the contribution of double doses to VTE development. This is blatant when comparing the genotypic frequency between cases and controls.

On the other hand, FGA did not show any association with VTE, which is supported by previous reports where haplotypes between FGA and fibrinogen gamma (FGG) were not associated with coronary events [44]. Nevertheless, a haplotype association (FGA/FGG) was found in ischemic stroke, carotid artery intima-medial thickness (IMT), and other CVD events [13, 45]. These discrepancies could be related to the major confounding factors: the genetic structure, which principally provokes spurious associations in case-control studies [31, 46, 47]. However, additional research is required to clear up these discrepancies.

Our data, as well as the findings by other studies, suggest that some STR alleles may be associated with different cardiovascular diseases; however, the functional mechanism is not clear [12, 48]. STRs are present in untranslated and promoter regions as well as within coding and intron regions where they represent 25% and 17%, respectively [19]. Also, there is evidence that these sequences act as transcription regulatory elements where the number and type of repeat units play a crucial role in modifying the level of transcription and, therefore, gene regulation even in neighbour genes [49–52]. Alternatively, STRs may also influence splicing patterns, mRNA processing, stability, and translation, thus affecting several protein functions [19, 53, 54]. All of these characteristics highlight the importance of studying STRs which have been associated with phenotypic consequences, cell surface variability, plasticity in skeletal morphology, and even drug susceptibility which could be related to other diseases [19]. Other reports provide compelling evidence that some CODIS markers (TH01, vWA, TPOX, and D21S11) could be associated with coronary heart disease, myocardial infarction, and increased heart weight, which is concordant with our findings [48, 55, 56]. Although STRs are good candidate markers that may contribute to understanding the origins of cardiovascular diseases, the combined effect of more genetic variants is more useful and notably contributes to finding biomarkers in complex diseases. Our study does not pretend to diminish the importance of other polymorphic markers such as SNPs, which have contributed, notably, to finding biomarkers in complex diseases. Hence, further research in other populations using SNPs and STRs is needed to identify additional genetic determinants. As well, haplotypes analyses with nearby SNPs would be informative, particularly, if variants of these proteins are likely to be causative of the phenotype. Studies in which the levels of von Willebrand factor A and thyroid peroxidase are measured and their relationship to different alleles estimated would also be desirable.

To our knowledge, this is the first study associating microsatellites with VTE in which the source of false positive association attributable to population stratification is avoided. However, some limitations of this study should be acknowledged, such as the sample size, which, despite being suitable to sample size calculations, only shows preliminary evidence of the relationship between VTE and the STRs analysed. Therefore, it is necessary to increase the sample size so as to obtain conclusive results about the association of these markers with VTE. Nonetheless, our study could be useful to define the size needed to determine a critical effect. Although all unprovoked VTE events were objectively documented and our calculations were adjusted taking into account some factors that may influence susceptibility to VTE such as age, gender, smoking status, and family history, our study had limitations in the body mass index, which was not followed by time; as a consequence, it was not documented objectively. As well, it is important to contemplate the effect of additional confounders on the VTE development.

In conclusion, the vWA and TPOX microsatellites could be good candidate biomarkers in venous thromboembolism diseases and could help to elucidate their origins. Additionally, these polymorphisms could become useful markers for genetic studies of VTE in the Mexican population; however, further studies should be done in order to confirm and validate this preliminary evidence.

## Supplementary Material

All genotypes as well as the differences in their frequencies between cases and controls are shown in Supplemental material.

## Figures and Tables

**Table 1:** Baseline information obtained from cases and nonrelated healthy individuals.

Variable	Females					Males				
	Cases	Controls	OR	95% CI	*P*	Cases	Controls	OR	95% CI	*P*
	*n* = 98	*n* = 239				*n* = 79	*n* = 292			
Age	44.68 ± 14.64	25.37 ± 13.20	1.11	1.110–1.113	≤0.0001	45.36 ± 15.94	30.80 ± 17.87	1.04	1.02–1.05	≤0.0001
Range	18–80	1 A 52				17–83	1 A 67			
Smoking habit										
Nonsmoking	78 (79.59)	171 (71.54)	1.51	0.82–2.77	0.15	46 (58.22)	146 (50.00)	1.40	0.82–2.39	0.18
Smoking	20 (20.41)	66 (27.61)				33 (41.77)	147 (50.34)			

OR: odds ratio; 95% CI: confidence intervals.

**Table 2:** Allelic frequencies and descriptive statistics of vWA, TPOX, and FGA loci in cases and nonrelated healthy individuals.

	Allelic frequencies					
Allele	vWA		TPOX		FGA	
	Cases	Controls	Cases	Controls	Cases	Controls
	*n* = 177	*n* = 531	*n* = 177	*n* = 531	*n* = 177	*n* = 531
6	—	—	0	0.0019	—	—
7	—	—	0.0056	0.0104	—	—
8	—	—	**0.1638**	**0.5075**	—	—
9	—	—	**0.3305**	**0.0642**	—	—
10	—	—	0.0791	0.05	—	—
11	0.0113	0	0.1554	0.2462	—	—
12	0.0056	0	**0.2062**	**0.1189**	—	—
13	0	0.0047	**0.0565**	**0.0009**	—	—
14	0.0678	0.0594	0.0028	0	—	—
15	0.0989	0.0972	—	—	—	—
15.2	0	0.0009	—	—	—	—
16	0.2994	0.3019	—	—	—	—
17	0.2486	0.3122	—	—	0.0085	0.0009
18	**0.2232**	**0.1396**	—	—	0.0028	0.0075
18.2	—	—	—	—	0	0 0.0009
19	0.0367	0.0764	—	—	0.0254	0.0774
19.2	—	—	—	—	0	0.0009
20	0.0085	0.0075	—	—	0.0932	0.084
21	—	—	—	—	0.113	0.1264
21.2	—	—	—	—	0	0.0038
22	—	—	—	—	0.1102	0.1311
23	—	—	—	—	0.1017	0.1387
23.2	—	—	—	—	0	0.0047
24	—	—	—	—	0.1356	0.1613
24.2	—	—	—	—	0	0.0047
25	—	—	—	—	0.1413	0.1491
25.2	—	—	—	—	0	0.0009
26	—	—	—	—	**0.1243 **	**0.0774**
26.2	—	—	—	—	0.0197	0.0019
27	—	—	—	—	**0.0876 **	**0.0217**
27.2	—	—	—	—	0	0.0009
28	—	—	—	—	**0.0311 **	**0.0057**
30.2	—	—	—	—	0.0056	0

	Descriptive Statistics					
*k *	9	9	8	8	14	20
Ho	0.6893	0.7887	0.5989	0.6321	0.8983	0.8792
He	0.7828	0.7741	0.7878	0.6619	0.8911	0.8797
*F*_IS_	0.122	−0.018	0.242	0.046	−0.005	0.002
HW (*P*)	**0.0005**	0.81	≤**0.0001**	0.0434	0.6162	0.4806

*k: *number of alleles; Ho: observed heterozygosity; He: expected heterozygosity; HW: Hardy-Weinberg (Weir and Cockerham *F *Statistic) *P  *value. Bold numbers indicate data with significant statistical differences.

**Table 3:** Statistical differences between cases and healthy controls.

Allelic frequency/loci	Cases	Controls	*P*_*a*_	*P*_*b*_	OR	95% CI
	*n* = 177	*n* = 531				
vWA-18	0.2232 (79)	0.1396 (148)	≤**0.0001**	<**0.05**	**1.77**	**1.29–2.43**
TPOX-9	0.3305 (117)	0.0642 (68)	≤**0.0001**	≤**0.0001**	**7.22**	**5.12–10.18**
TPOX-12	0.2062 (73)	0.1189 (126)	≤**0.0001**	≤**0.0001 **	**1.93**	**1.39–2.68**
TPOX-13	0.0560 (20)	0.0009 (1)	≤**0.0001**	>0.05	63.53	9.04–1276.35
FGA-26	0.1240 (44)	0.0770 (82)	**0.007**	>0.05	**1.7**	**1.13–2.54**
FGA-27	0.0880 (31)	0.0220 (23)	**0.0001**	>0.05	**4.34**	**2.41–7.81**
FGA-28	0.0310 (11)	0.0060 (6)	**0.0001**	>0.05	5.64	1.92–17.25

OR*: *odds ratio; 95% CI: confidence interval; *P*_*a*_: statistical differences without admixture correction; *P*_*b*_: statistical differences after admixture correction. Data are adjusted by Bonferroni correction (*P* ≤ 0.016). Significant statistical differences and accurate confidence intervals are shown in bold numbers.

**Table 4:** Statistical differences between risk allele doses in case and control populations.

Locus	Cases		Controls		*P *	OR (95% CI)
vWA	18, 18	18, X	18, 18	18, X	**0.0232**	**1.51 (1.04–2.20)**
	*n* = 14 (8%)	*n* = 50	*n* = 4 (0.75%)	*n* = 145		

TPOX	9, 9	9X	9, 9	9X	**< 0.0001**	**17.06 (11.07–26.36)**
	*n* = 31 (17.5%)	*n* = 89	*n* = 2 (0.37%)	*n* = 57		
	12, 12	12X	12, 12	12X	**< 0.0001**	**2.3 (1.6–3.3)**
	*n* = 31 (31%)	*N* = 47	*N* = 9 (12%)	*N* = 114		

X: any allele accompanying the risk allele; OR*: *odds ratio; 95% CI: confidence interval.

Significant statistical differences and accurate confidence intervals are shown in bold numbers.

**Table 5:** Contribution of candidate genes (alleles and genotypes) to venous thromboembolisms.

Locus	Allele/genotype	*P*	OR (95% CI)
vWA	18	**0.0001 **	**2.32 (1.53–3.53) **
	18, 18	**0.0001 **	**26.59 (6.90–102.48)**
	18, X	0.103	1.43 (0.93–2.21)

TPOX	9	**0.0001 **	**10.66 (6.45**–**17.62)**
	9, 9	**0.0001 **	**78.27 (18.29**–**42.59)**
	9, X	**0.0001 **	**4.88 (2.96**–**8.05)**
	12	0.057	1.51 (0.99–2.31)
	12, 12	**0.006 **	**4.22 (1.50**–**11.85) **
	12, X	0.443	1.19 (0.76–1.86)

Models were adjusted by age, gender, smoking habit, and family history. OR*: *odds ratio; 95% CI: confidence interval of logistic multiple regression analysis; X: any allele accompanying the risk allele.

Significant statistical differences and accurate confidence intervals are shown in bold numbers.

**Table 6:** Statistical differences between multiloci genotype frequencies in cases and controls (vWA, TPOX).

Multiloci genotypes	Frequency		*χ* ^2^	*P*	OR (95% CI)
(vWA, TPOX)	Cases	Controls			
18, 9	0.184	0.021	62.15	<**0.0001 **	**10.21 (4.93–21.49) **
18, 12	0.106	0.057	4.89	**0.0269 **	**1.94 (1.02–3.64) **

*χ* ^2^: Chi-square test; OR*: *odds ratio; 95% CI: confidence interval. Significant statistical differences and accurate confidence intervals are shown in bold numbers.
