# Supplementary material for: Association of vWA and TPOX Polymorphisms with Venous Thrombosis in Mexican Mestizos
Source: Biomed Res Int. 2014 Aug 31;2014:697689. doi: 10.1155/2014/697689 (PMC4164132; doi:10.1155/2014/697689)
Supplement: Supplementary file 1 — All genotypes as well as the differences in their frequencies between cases and controls are shown in Supplemental material. [file 697689.f1.pdf]

Supplemental Material

Genotypic statistical differences between cases and controls

**Locus**	**Genotypes**	**Cases**	**Controls**	***P***	**Odd's ratio ($_{95\%}$CI)**
	11,11	0.006 (1)	0	≥ 0.05	-
	11,12	0.011 (2)	0	≥ 0.05	-
	13,16	0	0.002 (1)	≥ 0.05	-
	13,17	0	0.006 (3)	≥ 0.05	-
	13,18	0	0.002 (1)	≥ 0.05	-
	14,14	0	0.009 (5)	≥ 0.05	-
	14,15	0.006 (1)	0.023 (12)	≥ 0.05	0.25 (0.01 - 1.83)
	14,16	0.073 (13)	0.026 (14)	**0.004**	**2.93 (1.27 - 6.76)**
	14,17	0.011 (2)	0.034 (18)	≥ 0.05	0.33 (0.05 - 1.48)
	14,18	0.034 (6)	0.009 (5)	**0.03**	3.69 (0.98 - 14.12)
	14,19	0.006 (1)	0.006 (3)	≥ 0.05	1
	14,20	0	0.002 (1)	≥ 0.05	-
	15,15	0.028 (5)	0.006 (3)	**0.03**	5.12 (1.06 -27.26)
	15,16	0.051 (9)	0.064 (34)	≥ 0.05	0.78 (0.34 - 1.74)
	15,17	0.062 (11)	0.058 (31)	≥ 0.05	1.07 (0.49 - 2.27)
**vWA**	15,18	0.023 (4)	0.026 (14)	≥ 0.05	0.85 (0.23 - 2.82)
	15,19	0	0.011 (6)	≥ 0.05	-
	15.2, 17	0	0.002 (1)	≥ 0.05	-
	16,16	0.102 (18)	0.087 (46)	≥ 0.05	1.19 (0.65 - 2.19)
	16,17	0.153 (27)	0.194 (103)	≥ 0.05	0.75 (0.46 - 1.22)
	16,18	0.113 (20)	0.105 (56)	≥ 0.05	1.08 (0.61 - 1.91)
	16,19	0.017 (3)	0.036 (19)	≥ 0.05	0.46 (0.11 - 1.68)
	16,20	0	0.006 (3)	≥ 0.05	-
	17,17	0.085 (15)	0.090 (48)	≥ 0.05	0.93 (0.48 - 1.77)
	17,18	0.079 (14)	0.092 (49)	≥ 0.05	0.75 (0.38 - 1.45)
	17,19	0.017 (3)	0.053 (28)	**0.043**	0.31 (0.07 - 1.08)
	17,20	0.011 (2)	0.004 (2)	≥ 0.05	3.02 (0.30 - 30.18)
	18,18	0.079 (14)	0.008 (4)	**≤ 0.0001**	11.32 (3.42 - 41. 29)
	18,19	0.023 (4)	0.028 (15)	≥ 0.05	0.80 (0.22 - 2.60)
	18,20	0.006 (1)	0	≥ 0.05	-
	19,19	0.006 (1)	0.009 (5)	≥ 0.05	0.60 (0.03 - 5.28)
	20,20	0	0.002 (1)	≥ 0.05	-

Locus	Genotypes	Cases	Controls	*P*	Odd's ratio ($_{95\%}$CI)
	6,8	0	0.002 (1)	≥ 0.05	-
	6, 9.3	0	0.002 (1)	≥ 0.05	-
	7,7	0	0.002 (1)	≥ 0.05	-
	7,8	0	0.008 (4)	≥ 0.05	-
	7,9	0.011 (2)	0.006 (3)	≥ 0.05	2.01 (0.23- 14.87)
	7,10	0	0.002 (1)	≥ 0.05	-
	7,11	0	0.002 (1)	≥ 0.05	-
	8,8	0.073 (13)	0.275 (146)	**≤ 0.0001**	**0.21 (0.11 - 0.39)**
	8,9	0.034 (6)	0.060 (32)	≥ 0.05	0.55 (0.20 - 1.40)
	8,10	0.034 (6)	0.047 (25)	≥ 0.05	0.71 (0.26 - 1.86)
	8,11	0.090 (16)	0.224 (119)	**≤ 0.0001**	**0.34 (0.19 - 0.61)**
	8,12	0.017 (3)	0.122 (65)	**≤ 0.0001**	0.12 (0.03 - 0.41)
	8,13	0.006 (1)	0.002 (1)	≥ 0.05	3.01 (0 - 110.59)
**TPOX**	9,9	0.175 (31)	0.004 (2)	**≤ 0.0001**	56.16 (12.90 - 343.46)
	9,10	0.062 (11)	0.008 (4)	**≤ 0.0001**	8.73 (2.53 - 32.96)
	9,11	0.034 (6)	0.038 (20)	≥ 0.05	0.90 (0.32 - 2.41)
	9,12	0.147 (26)	0.009 (5)	**≤ 0.0001**	18.11 (6.47 - 54.73)
	9,13	0.023 (4)	0	≥ 0.05	-
	9,14	0.006 (1)	0	≥ 0.05	-
	10,10	0.006 (1)	0.004 (2)	≥ 0.05	1.50 (-)
	10,11	0.011 (2)	0.024 (13)	≥ 0.05	0.46 (0.07 - 2.14)
	10,12	0.028 (5)	0.011 (6)	≥ 0.05	2.54 (0.67 - 9.54)
	10,13	0.011 (2)	0	≥ 0.05	-
	11,11	0.068 (12)	0.070 (37)	≥ 0.05	0.97 (0.47 - 1.98)
	11,12	0.028 (5)	0.064 (34)	≥ 0.05	0.42 (0.14 - 1.16)
	11,13	0.011 (2)	0	≥ 0.05	-
	12,12	0.073 (13)	0.015 (8)	**≤ 0.0001**	**5.18 (1.97 - 13.93)**
	12,13	0.040 (7)	0	≥ 0.05	-
	13,13	0.011 (2)	0	≥ 0.05	-

Locus	Genotypes	Cases	Controls	*P*	Odd’s ratio ($_{95\%}$CI)
	17,20	0.006 (1)	0	≥ 0.05	-
	17,21	0	0.002 (1)	≥ 0.05	-
	17,26	0.006 (1)	0	≥ 0.05	-
	17,27	0.006 (1)	0	≥ 0.05	-
	18,18	0	0.002 (1)	≥ 0.05	-
	18,19	0	0.004 (2)	≥ 0.05	-
	18,20	0.006 (1)	0	≥ 0.05	-
	18,21	0	0.002 (1)	≥ 0.05	-
	18,23	0	0.002 (1)	≥ 0.05	-
	18,26	0	0.002 (1)	≥ 0.05	-
	18,27	0	0.002 (1)	≥ 0.05	-
	18.2,22	0	0.002 (1)	≥ 0.05	-
	19,19	0	0.002 (1)	≥ 0.05	-
	19,20	0	0.015 (8)	≥ 0.05	-
	19,21	0.011 (2)	0.023 (12)	≥ 0.05	0.49 (0.08 - 2.35)
	19,22	0.006 (1)	0.019 (10)	≥ 0.05	0.30 (0.01 - 2.26)
	19,23	0.006 (1)	0.023 (12)	≥ 0.05	0.25 (0.01 - 1.83)
	19,23.2	0	0.002 (1)	≥ 0.05	-
	19,24	0.023 (4)	0.023 (12)	≥ 0.05	1.00 (0.27 - 3.39)
	19,25	0	0.019 (10)	≥ 0.05	-
	19,26	0	0.017 (9)	≥ 0.05	-
	19,27	0.006 (1)	0.008 (4)	≥ 0.05	0.75 (0.03 - 7.12)
	19.2,22	0	0.002 (1)	≥ 0.05	-
	20,20	0.006 (1)	0.006 (3)	≥ 0.05	1.00 (-)
	20,21	0.028 (5)	0.030 (16)	≥ 0.05	0.94 (0.30 - 2.77)
	20,22	0.017 (3)	0.017 (9)	≥ 0.05	1.00 (0.21 - 4.07)
	20,23	0.017 (3)	0.023 (12)	≥ 0.05	0.75 (0.17 - 2.87)
	20,24	0.017 (3)	0.028 (15)	≥ 0.05	0.59 (0.13 - 2.21)
	20,25	0.045 (8)	0.030 (16)	≥ 0.05	**1.52 (0.59 -3.86)**
	20,26	0.028 (5)	0.013 (7)	≥ 0.05	2.18 (0.59 - 7.74)
	20,27	0.006 (1)	0	≥ 0.05	-
	20,28	0.006 (1)	0	≥ 0.05	-
	21,21	0.006 (1)	0.009 (5)	≥ 0.05	0.60 (0.03 -5.28)
	21,22	0.023 (4)	0.034 (18)	≥ 0.05	0.66 (0.19 - 2.10)
	22,23	0.017 (3)	0.028 (15)	≥ 0.05	0.59 (0.13 - 2.21)
	21,24	0.034 (6)	0.045 (24)	≥ 0.05	0.74 (0.27 - 1.95)
	21,25	0.045 (8)	0.041 (22)	≥ 0.05	1.10 (0.44 - 2.65)
	21,26	0.028 (5)	0.021 (11)	≥ 0.05	1.37 (0.41 - 4.35)
	21,27	0.023 (4)	0.006 (3)	**0.048**	4.07 (0.76 - 21.09)
	21,28	0	0.002 (1)	≥ 0.05	-
**FGA**	21,30.2	0.006 (1)	0	≥ 0.05	-
	21.2, 23.2	0	0.002 (1)	≥ 0.05	-
	21.2,24	0	0.002 (1)	≥ 0.05	-
	21.2,24.2	0	0.002 (1)	≥ 0.05	-
	21.2,26	0	0.002 (1)	≥ 0.05	-
	22,22	0.011 (2)	0.024 (13)	≥ 0.05	0.46 (0.07 - 2.14)
	22,23	0.023 (4)	0.036 (19)	≥ 0.05	0.62 (0.18 - 1.97)
	22,24	0.040 (7)	0.060 (32)	≥ 0.05	0.64 (0.25 - 1.56)
	22,25	0.028 (5)	0.034 (18)	≥ 0.05	0.83 (0.27 - 2.41)
	22,26	0.034 (6)	0.008 (4)	**0.01**	4.62 (1.14 - 19.72)
	22,27	0.028 (5)	0.002 (1)	**0.0009**	15.41 (1.75 - 350.92)
	23,23	0.017 (3)	0.026 (14)	≥ 0.05	0.64 (0.14 - 2.40)
	23,23.2	0	0.002 (1)	≥ 0.05	-
	23,24	0.023 (4)	0.032 (17)	≥ 0.05	0.52 (0.12 - 1.92)
	23,25	0.051 (9)	0.041 (22)	≥ 0.05	**1.24 (0.52 - 2.89)**
	23,26	0.006 (1)	0.034 (18)	**0.044**	0.16 (0.01 - 1.16)
	23,27	0.028 (5)	0.004 (2)	**0.004**	7.69 (1.31 - 57.70)
	23,28	0	0.002 (1)	≥ 0.05	-
	23.2,24	0	0.002 (1)	≥ 0.05	-
	23.2,26	0	0.002 (1)	≥ 0.05	-
	24,24	0.028 (5)	0.023 (12)	≥ 0.05	1.26 (0.38 - 3.91)
	24,25	0.023 (4)	0.051 (27)	≥ 0.05	0.43 (0.13 - 1.32)
	24,26	0.040 (7)	0.021 (11)	≥ 0.05	**1.95 (0.67 - 5.52)**
	24,26.2	0.006 (1)	0.002 (1)	≥ 0.05	3.01 (0.00 - 110.59)
	24,27	0.006 (1)	0.008 (4)	≥ 0.05	0.75 (0.03 - 7.12)
	24,28	0.006 (1)	0.004 (2)	≥ 0.05	1.50 (-)
	24.2,25.2	0	0.002 (1)	≥ 0.05	-
	24.2,26	0	0.002 (1)	≥ 0.05	-
	24.2,26.2	0	0.002 (1)	≥ 0.05	-
	24.2,27.2	0	0.002 (1)	≥ 0.05	-
	25,25	0	0.024 (13)	≥ 0.05	-
	25,26	0.034 (6)	0.024 (13)	≥ 0.05	1.40 (0.47 - 4.02)
	25,26.2	0.017 (3)	0	≥ 0.05	-
	25,27	0.040 (7)	0.008 (4)	**0.002**	5.43 (1.41 - 22.31)
	25,28	0.011 (2)	0.002 (1)	≥ 0.05	6.06 (0.43 - 169.64)
	26,26	0.017 (3)	0.002 (1)	**0.02**	9.14 (0.85 - 229.37)
	26,27	0.017 (3)	0.004 (2)	≥ 0.05	4.56 (0.62 - 39.25)
	26,28	0.017 (3)	0.002 (1)	**0.02**	9.14 (0.85 - 229.37)
	26,30.2	0.006 (1)	0	≥ 0.05	-
	26.2,27	0.006 (1)	0	≥ 0.05	-
	27,27	0.006 (1)	0.002 (1)	≥ 0.05	3.01 (0.00 - 110.59)
	28,28	0.011 (2)	0	≥ 0.05	-
